# The German research consortium for the study of bipolar disorder (BipoLife): a quality assurance protocol for MR neuroimaging data

**DOI:** 10.1186/s40345-024-00354-7

**Published:** 2024-09-26

**Authors:** Christoph Vogelbacher, Jens Sommer, Miriam H. A. Bopp, Irina Falkenberg, Philipp S. Ritter, Felix Bermpohl, Catherine Hindi Attar, Karolin E. Einenkel, Oliver Gruber, Georg Juckel, Vera Flasbeck, Martin Hautzinger, Andrea Pfennig, Silke Matura, Andreas Reif, Dominik Grotegerd, Udo Dannlowski, Tilo Kircher, Michael Bauer, Andreas Jansen

**Affiliations:** 1 Department of Psychology, Philipps University of Marburg, Marburg, Germany; 2 Center for Mind, Brain and Behavior (CMBB), University of Marburg and Justus Liebig University Giessen, Giessen, Germany; 3 Core-Facility Brainimaging, Faculty of Medicine, University of Marburg, Marburg, Germany; 4 Department of Neurosurgery, University of Marburg, Marburg, Germany; 5 Department of Psychiatry and Psychotherapy, University of Marburg, Marburg, Germany; 6 Department of Psychiatry and Psychotherapy, Carl Gustav Carus University Hospital, Technische Universität Dresden, Dresden, Germany; 7 Department of Psychiatry and Clinical Neuroscience, Campus Charité Mitte, Berlin, Germany; 8 St. Hedwig Hospital, Charité - Universitätsmedizin Berlin, Berlin, Germany; 9 Corporate Member of Freie Universität Berlin, Humboldt-Universität Zu Berlin, and Berlin Institute of Health, Berlin, Germany; 10 Section for Experimental Psychopathology and Neuroimaging, Department of General Psychiatry, Heidelberg University, Heidelberg, Germany; 11 Department of Psychiatry, Psychotherapy and Preventive Medicine, LWL University Hospital, Ruhr-University, Bochum, Germany; 12 Department of Psychology Clinical Psychology and Psychotherapy, Eberhard Karls University, Tübingen, Germany; 13 Department of Psychiatry, Psychosomatic Medicine and Psychotherapy, Goethe University Frankfurt, University Hospital, Frankfurt, Germany; 14 Fraunhofer Institute for Translational Medicine and Pharmacology ITMP, Frankfurt, Germany; 15 Institute for Translational Psychiatry, University of Münster, Münster, Germany

**Keywords:** Bipolar disorder, MRI, Quality assurance, Multicenter study, Early recognition, Early intervention, BipoLife

## Abstract

**Background:**

The German multicenter research consortium BipoLife aims to investigate the mechanisms underlying bipolar disorders. It focuses in particular on people at high risk of developing the disorder and young patients in the early stages of the disease. Functional and structural magnetic resonance imaging (MRI) data was collected in all participating centers. The collection of neuroimaging data in a longitudinal, multicenter study requires the implementation of a comprehensive quality assurance (QA) protocol. Here, we outline this protocol and illustrate its application within the BipoLife consortium.

**Methods:**

The QA protocol consisted of (1) a training of participating research staff, (2) regular phantom measurements to evaluate the MR scanner performance and its temporal stability across the course of the study, and (3) the assessment of the quality of human MRI data by evaluating a variety of image metrics (e.g., signal-to-noise ratio, ghosting level). In this article, we will provide an overview on these QA procedures and show exemplarily the influence of its application on the results of standard neuroimaging analysis pipelines.

**Discussion:**

The QA protocol helped to characterize the various MR scanners, to record their performance over the course of the study and to detect possible malfunctions at an early stage. It also assessed the quality of the human MRI data systematically to characterize its influence on various analyses. Furthermore, by setting up and publishing this protocol, we define standards that must be considered when analyzing data from the BipoLife consortium. It further promotes a systematic evaluation of data quality and a definition of subject inclusion criteria. In the long term, it will help to increase the chance of achieving clinically relevant results.

**Supplementary Information:**

The online version contains supplementary material available at 10.1186/s40345-024-00354-7.

## Introduction

Bipolar disorder (BD) is a severe, recurrent and heterogeneous mental disorder. It affects more than 1% of the population worldwide and typically has its onset during youth. Its chronic course is associated with high rates of morbidity and mortality, a high risk of suicide, and poor social and occupational outcomes, making it one of the main causes of disability among young and working-age people worldwide (Bauer 2022; GBD 2019 Mental Disorders Collaborators 2022; McIntyre et al. 2020; Saraf et al. 2021). Despite the great advances over the last decades in understanding mental disorders, the mechanisms underlying BD at the cellular, transmitter and neural network level still remain elusive. This has severe consequences for clinical practice.

The German research consortium BipoLife, established in 2015, aims to investigate the basis and causes of BD. It focuses on the one hand on the prevention, diagnosis and therapeutic intervention for patients, on the other hand on the identification of biomarkers. The consortium comprises, among others, several multicenter clinical studies, a naturalistic-epidemiological study and basic neuroscientific research projects (for a detailed description of the consortium, see Pfennig et al. 2020; Ritter et al. 2016). In this network, functional and structural magnetic resonance imaging (MRI) data were acquired in several projects (A1, A2 and B2) across nine neuroimaging centers using a standardized neuroimaging protocol [for a detailed overview on the MRI study protocol, acquisition parameters and the neuroimaging paradigms, see Vogelbacher et al. (2021)].

The MR image characteristics can change significantly over the course of a longitudinal study and strongly differs between MR scanners, even if scanners from the same manufacturer are used (Friedman and Glover 2006; Vogelbacher 2020). In particular, the temporal stability of the data is crucial in order to be able to map possible changes caused by the course of the disease. The acquisition of MRI data from up to nine centers therefore necessitates the standardization of the acquisition protocol and the implementation of regular quality assurance (QA) procedures to both detect potential MR scanner malfunctions at an early stage and to evaluate the quality of the acquired MRI data. The implementation of and in particular the adherence to a well-defined quality assurance protocol is considered as a key benchmark in the evaluation of the overall quality of multicenter imaging studies and its impact on the patient level (Glover et al. 2012; Van Horn and Toga 2009). Previously, it has been shown that the inclusion of low-quality MRI data can strongly influence the outcome of data analysis pipelines (e.g., Stöcker et al. 2005; Vogelbacher 2020).

Some form of QA protocol is used in most neuroimaging centers, although sometimes it is just the standard manufacturer’s protocol that is carried out during regular maintenance. Often, these protocols are explicitly described only in the context of large-scale multicenter studies (e.g., the FBIRN consortium), but not in smaller single site studies. Depending on the main questions, they may focus on different aspects of the MRI data, e.g., signal-to-noise ratio (SNR), image homogeneity, differentiation between tissue classes or, especially in the context of functional MRI (fMRI) studies, temporal stability [for an overview on the set-up of QA protocols for MR scanners, see Sreedher et al. (2021); for an in-depth review on previous studies which developed QA programs, including QA phantoms, QA metrics and preprocessing pipelines, see Lu et al. (2019)].

In the BipoLife consortium, we implemented a comprehensive QA protocol for the acquisition of MRI data. This protocol consisted of (i) a thorough training of participating research staff, (ii) regular phantom measurements to evaluate the MR scanner performance and to assess the impact of changes in scanner hardware and software, and (iii) the assessment of the quality of human MRI data by evaluating a variety of image metrics (e.g., SNR, ghosting level). In this article, we will provide an overview on these QA procedures, show their applicability and demonstrate exemplarily that for instance the SNR has an influence on structural MRI data results. This article therefore complements the previously published description of the BipoLife MRI study protocol (Vogelbacher et al. 2021).

## Methods

The BipoLife consortium involved nine neuroimaging centers across Germany. All data were acquired on 3 Tesla MR scanners. These scanners were from different manufacturers and had different hard- and software configurations (e.g., different head coils). MR sequence parameters were standardized across all sites to the extent permitted by each scanner. All subjects were assessed both with a high resolution T1-weighted anatomical image and several functional measurements (three task-based fMRI paradigms, one resting-state measurement). Additionally, a phantom was measured after each subject. We specifically decided to measure the phantom not at the beginning but at the end, i.e., after the human MRI data was acquired. This ensured that the MR scanner was in an almost comparable condition each time. We have to acknowledge that the acquisition protocol is EPI heavy. When the scanning session is completed, the gradients might be hot, potentially leading to artifacts (e.g., increased drifts) that had not yet occurred when the human MRI data was acquired. For future studies, one might therefore think about counter-balancing the order of phantom acquisitions. An extensive description of the MR scanners, their hardware configurations and software packages can be found in Table 1 [of note, the MR sequence parameters and the experimental design can be found elsewhere (Vogelbacher et al. 2021)].

**Table 1:** MR scanners, their hardware configurations and software packages used in the BipoLife consortium

Site	Manufacturer	MR scanner type	Field strength	Receive coil	Software changes	Software version (old > new)
BE	Siemens	Tim Trio	3 Tesla	20 channels	No	syngo MR B17
BO	Philips	Achieva	3 Tesla	32 channels	Yes	5.1.7\5.1.7.2 > 5.3.1\5.3.1.1
DR	Siemens	Tim Trio	3 Tesla	12 channels	No	syngo MR B17
FR	Siemens	Trio	3 Tesla	8 channels	No	syngo MR A35 4VA35A
GÖ	Siemens	Tim Trio	3 Tesla	12 channels	No	syngo MR B17
HA	Siemens	Skyra	3 Tesla	32 channels	Yes	syngo MR D13 > syngo MR E11
MA	Siemens	Tim Trio	3 Tesla	12 channels	No	syngo MR B17
TÜ	Siemens	Prisma	3 Tesla	20 channels	Yes	syngo MR D13 > syngo MR E11

*BE* Berlin, *BO* Bochum, *DR* Dresden, *FR* Frankfurt, *GÖ* Göttingen, *HA* Hamburg, *MA* Marburg, *TÜ* Tübingen

Of note the University of Heidelberg was included as 9th center in the Bipolife consortium. Since only one MRI data set was acquired at this site (for project B2) we decided to exclude this data

After the study protocol was set up at all centers, the participating sites were visited, the local staff was trained and the compliance with the study protocol was verified. Each center performed a complete measurement of one subject in the presence of the coordinating team to clarify all open questions. Each center then carried out three further measurements on control subjects over the next few days to become familiar with the study protocol (e.g., preparation and execution of the MRI measurement, measurement of phantom data, data transfer to the coordinating center). The data was sent to the coordinating center. If there were no further objections, the study measurements could begin. Of note, for organizational reasons we did not have the possibility to measure the same subjects at each site (“traveling subjects”). This must be considered as a missed opportunity to further establish inter-scanner reliability. We strongly recommend future studies to also include these measurements.

During the project, all MRI data was sent via the internet directly after the measurement to the coordinating center. The transferred data was inspected at the coordinating center for potential errors in data acquisition. The inspection included a check of data completeness [with regard to human and phantom data, the logfiles and the clinical report form (CRF)] as well as a check of the correct positioning of the bounding box during the planning of the measurement. If local staff changed, new team members received thorough training from the coordination center.

Quality assurance was carried out on two levels. First, we used phantom data to evaluate the temporal stability of the MR scanners across time. Second, we assessed the quality of human MRI data using a variety of metrics. In the following, we will first give a detailed overview on the QA protocol for phantom MRI data (Section "QA protocol: Phantom MRI data") and for human MRI data (Section "QA protocol: Human MRI data"). We will then show that the data quality of structural human MRI data (high SNR vs. low SNR) can have a profound impact on the outcome of standard neuroimaging analysis pipelines (Section "Influence of the quality of MRI data on brain imaging analyses").

### QA protocol: phantom MRI data

In this section, we describe the measurement and analysis of phantom MRI data (Section "Assessment of the quality of phantom MRI data"), show how the results of the phantom measurements can be used to characterize the various properties of MR scanners (Section "Properties of different MR scanners") and how these properties can change over time (Section "Temporal stability of MR scanners"). This information can be used both to exclude specific data due to poor quality and to determine long-term changes in the quality of an MR scanner (e.g., after technical changes, Section "Influence of major technical changes at MR scanners").

#### Assessment of the quality of phantom MRI data

The phantom was a 23.5 cm long and 11.1 cm-diameter cylindrical plastic vessel (Rotilabo, Carl Roth GmbH+Co. KG, Karlsruhe, Germany) filled with a mixture of 62.5 g agar and 2000 ml distilled water. In contrast to widely used water filled phantoms, agar phantoms are more suitable for fMRI studies. On the one hand, T2 values and magnetization transfer characteristics are more similar to brain tissue (Hellerbach 2013), on the other hand they are less vulnerable to scanner vibrations and thus avoid a long settling time prior to data acquisition (Friedman and Glover 2006).

Phantom data was acquired after each subject measurement except when two subjects were measured consecutively. In this case, the MRI phantom was measured only once in between the two measurements. Alignment of the phantom was lengthwise, parallel to the z-axis, and at the center of the head coil. The alignment of the phantom was evaluated by the radiographer performing the measurement and—if necessary—corrected using the localizer scan. The positioning of the bounding box during the planning of the measurement was manually centered at the phantom with slice direction perpendicular to the phantom body (see supplementary material S1 for more details).

We decided to apply a T2*-weighted echo planar imaging (EPI) sequence because we were most interested in assessing the temporal stability of the MR scanners across fMRI measurements. We applied the same MR sequence parameters as in the resting-state measurement. The first 5 images were discarded from all analyses to account for equilibrium effects.

Various QA metrics can be calculated from phantom data, assessing for instance the strength of the signal, temporal stability and geometric distortions [for an overview, see Glover et al. (2012); Lu et al. (2019)]. We used QA metrics that covered various spatial and temporal aspects of the images, including the SNR, spatial inhomogeneity, ghosting artifacts, temporal fluctuations and scanner drift [the detailed mathematical description is presented in a previous publication of our research group (Vogelbacher et al. 2018)]. Data analysis was performed using the self-developed LAB-QA2GO software package (Vogelbacher et al. 2019).

Data acquisition started in October 2015 and ended in December 2020. Until this date, 431 phantom measurements were performed, of which 426 data sets were complete and could be further analyzed (some data had to be excluded based on a misplacement of the phantom; for an overview, see Table 2).

**Table 2:** Number of phantom measurements for each center

Center	BE	BO	DR	FR	GÖ	HA	MA	TÜ
Phantom measurements	116	10	53	47	8	55	90	52
Complete data sets	114	10	53	47	8	54	90	50

*BE *Berlin, *BO* Bochum, *DR* Dresden, *FR* Frankfurt, *GÖ* Göttingen, *HA* Hamburg, *MA* Marburg, *TÜ* Tübingen

#### Properties of different MR scanners

**Fig. 1:**
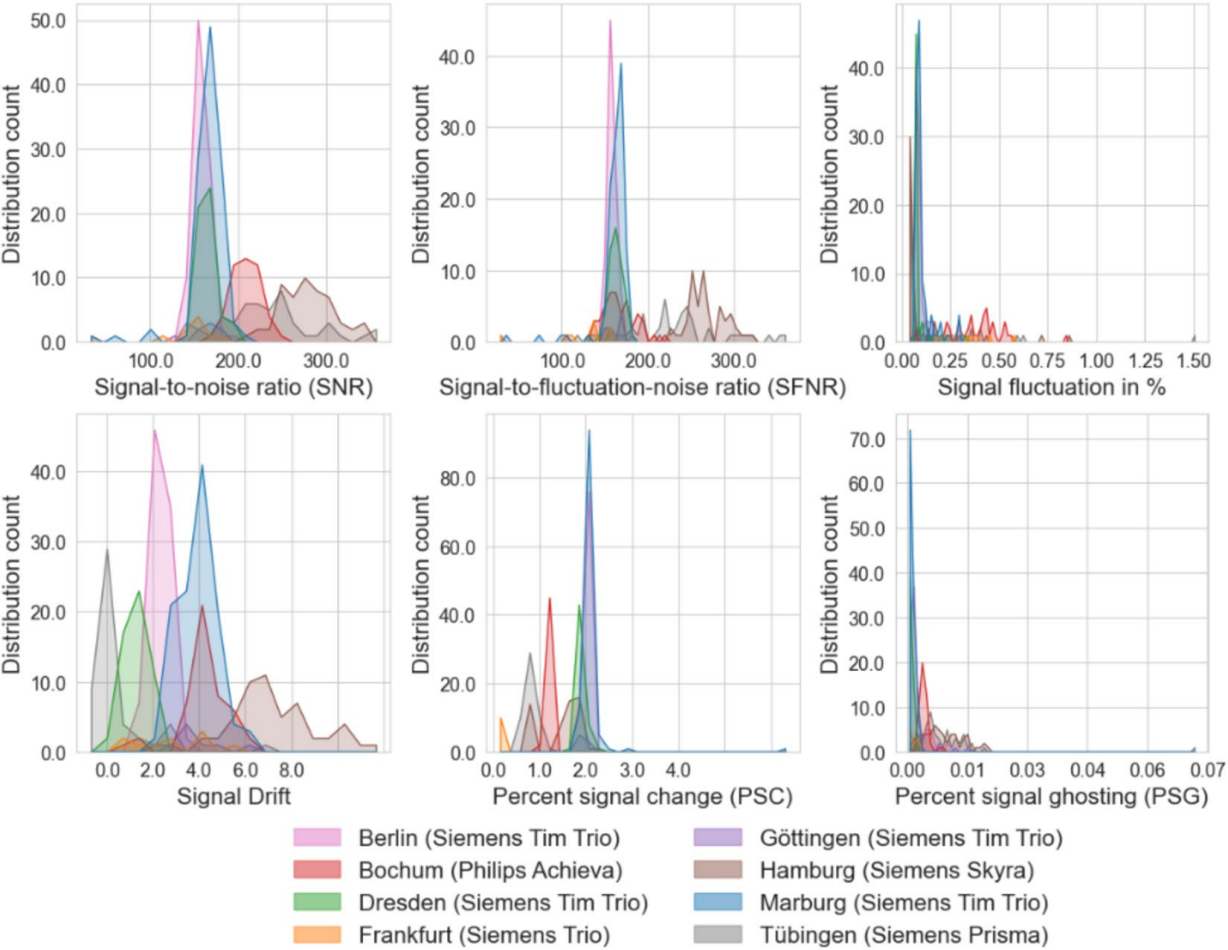
Overview over the distribution of various QA metrics for the phantom data. The overall performance, characterized by the QA metrics, strongly differs between the MR scanners, even between identical models. *SNR* signal-to-noise ratio, *SFNR* signal-to-fluctuation-noise ratio, *PSC* percent signal change, *PSG* percent signal ghosting [for details, see Vogelbacher (2020)]

In Fig. 1, we present the results of phantom QA analyses for each center (see also Supplement S2 for detailed values). It is clearly evident that there are clear differences in each QA metric between the different MR scanners, both in mean and variance. While it might be assumed that all MR scanners of the same type share approximately the same characteristics, in reality, there are significant differences, even among relatively similar models (see Table 1 for an overview of the used scanner). The typical SNR values for the MR scanners in Berlin, Dresden and Marburg (all using a Siemens Tim Trio), for instance, are relatively similar, while the other scanners clearly differ. However, these MR scanners also strongly differ in other metrics (e.g., drift values). Thus, the overall behavior of each MR scanner, characterized by the QA metrics, is unique.

#### Temporal stability of MR scanners

**Fig. 2:**
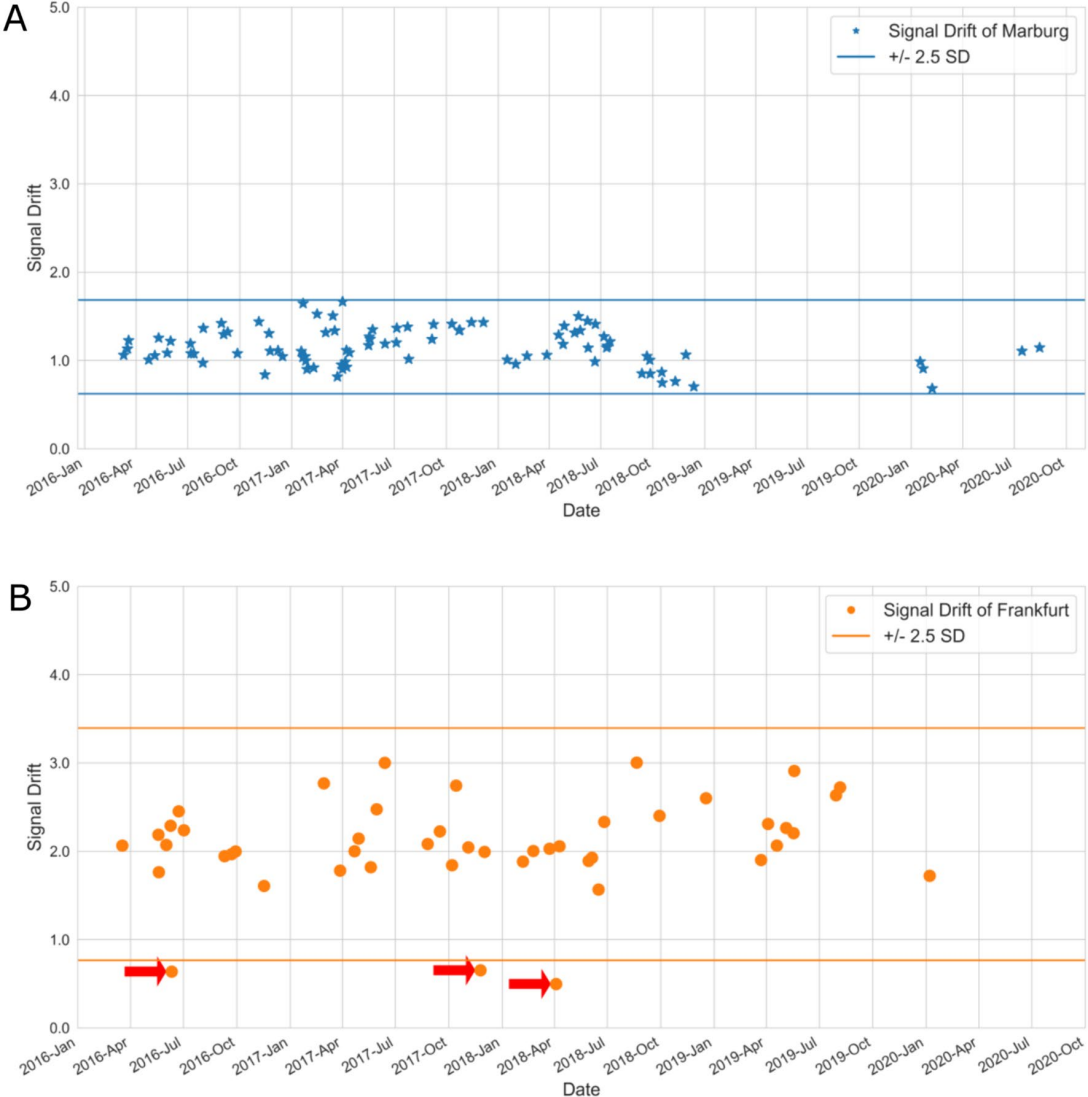
The scanner drift value for the centers of Marburg (**A**) and Frankfurt (**B**) over time. For a post-hoc outlier detection, we defined a range of ±2.5 standard deviations (*horizontal lines*). For Frankfurt, three outliers were identified (*red arrows*). A more detailed analysis showed however, that these deviations were caused by a wrong placement of the phantom, not by MR scanner malfunctions

In Fig. 2, we show how the scanner drift, as a specific example of a QA metric, developed over time, i.e., over the course of the study, at the Centers of Marburg and Frankfurt. Marburg shows in general a lower drift in comparison to the Frankfurt site. Also the overall variability across time is lower [coefficient of variation (CV) of Marburg = 0.18; CV of Frankfurt = 0.25]. In Table 3, we additionally show a year-by-year comparison of the variability of the drift for both centers. We believe that this year-by-year representation of QA metrics can help to better identify long-term trends in the change of MR scanner characteristics.

**Table 3:** Year-by-year comparison of the variability of the scanner drift for the Centers of Marburg and Frankfurt

	Scanner drift per year				
Center	2016	2017	2018	2019	2020
Marburg	0.13	0.18	0.20	–*	0.17
Frankfurt	0.23	0.26	0.29	0.14	–**

*In 2019, no measurements were performed in Marburg due to the pandemic

**In 2020, only one measurement was performed in Frankfurt, making it impossible to calculate a CV

Based on the fluctuation of this data, we can automatically calculate limit values [e.g., ±2.5 SD of the mean as a possible outlier (Friedman and Glover 2006)] and thus assess whether a QA metric deviates too much from the usual values and thus possibly indicates impairments in the function of the MR scanner. We would like to note that the limit values are arbitrary. If we had chosen smaller limits, we simply had to recheck more data points.

In Fig. 2, we used, for illustrative purposes, as limit a range of 2.5 standard deviations of the mean (based on all data points acquired during the study) for a post-hoc outlier detection. For Marburg, all measured values were within the permitted fluctuation range. For Frankfurt, one could detect three outliers (marked with red arrow). A closer inspection of the data, however, showed that these fluctuations were not caused by changes of scanner performance, but were related to a changed placement of the phantom in the MR scanner (placement differences are highlighted in supplementary material S3). A systematic and, most important, timely assessment of all QA values can be helpful in some cases to early detect potential scanner malfunctions (as described in Section "Influence of major technical changes at MR scanners" below). For future measurement, we recommend on the one hand to use a phantom holder to make sure that the placement of the phantom is as reliable as possible. On the other hand, we recommend calculating the QA metrics not only for one selected slice, but for a larger volume.

#### Influence of major technical changes at MR scanners

**Fig. 3:**
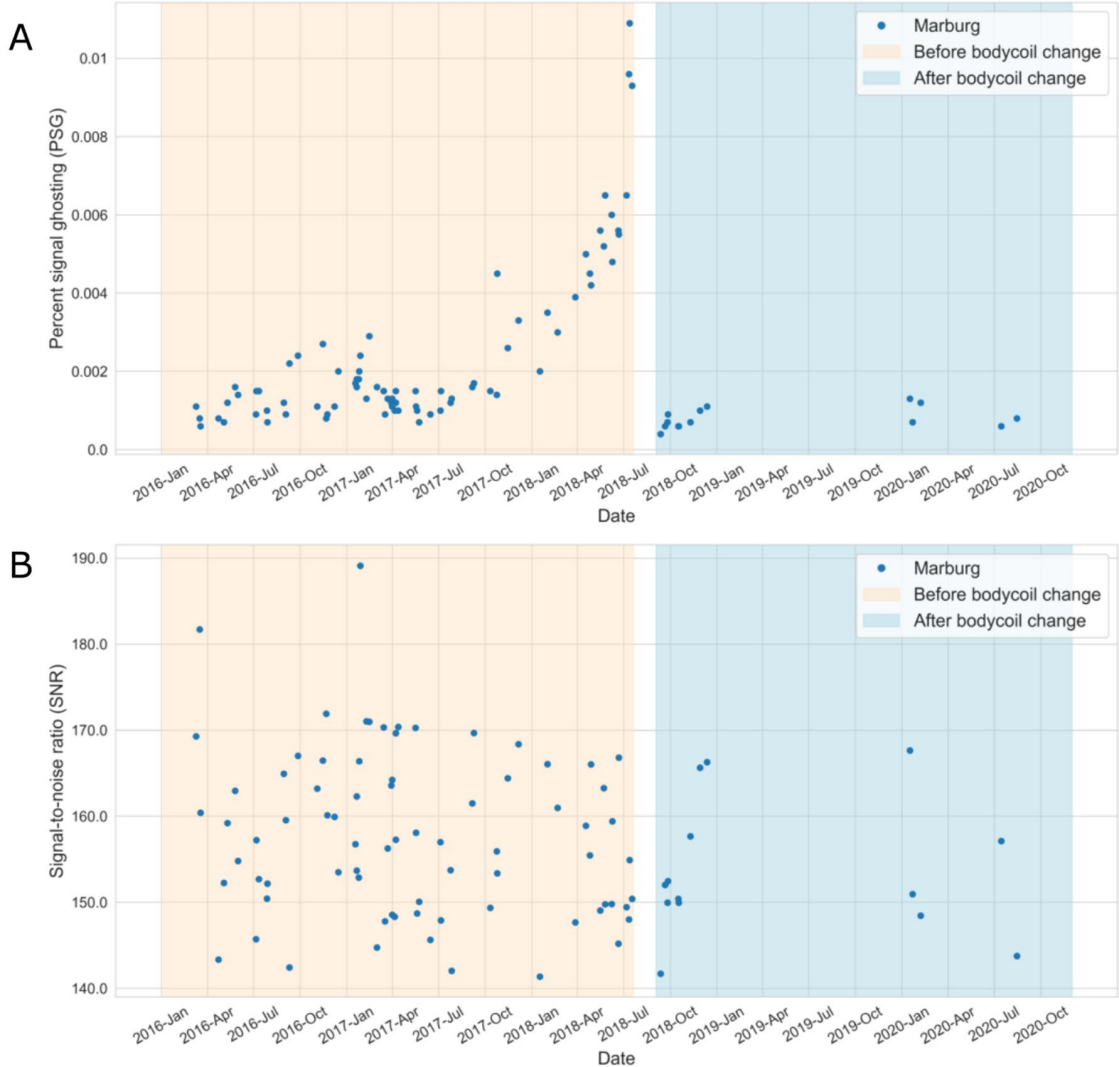
Percent signal ghosting (PSG) (**A**) and signal-to-noise ratio (SNR) (**B**) for the center of Marburg over time. The bisque area describes measurements before and the lightblue after the body coil was changed. No measurements took place between 17 June 2018 and 11 September 2018. The scanner shows in general a stable performance before and after replacement of the body coil, as indicated by stable QA values in all metrics calculated from the phantom data. There was an almost tenfold increase in PSG values before the body coil had to be replaced. If this had been noticed earlier, the scanner defect might have been detected sooner

The QA metrics are sensitive to technical changes of a scanner (such as the replacement of the MRI gradient coil), changes of the QA protocol (e.g., the introduction of special phantom holders) or changes of MR sequence parameters. In Fig. 3, we show an example of how damage to the body coil at the Marburg site affected the QA metrics and also how functional failures could have been detected in advance. In June 2018, there was a sudden failure of the MR scanner in Marburg. After extensive error diagnostics, the service technicians detected a defect of the body coil. After its replacement, the MRI system was working properly again. In a post-hoc analysis performed after the incident, we noticed that in a time interval of about two months before the failure of the MR scanner, the metrics assessing ghosting artifacts in the MR images [such as “percent-signal-ghosting”, PSG, cf. Vogelbacher et al. (2018)] were strongly increasing (Fig. 3, top). If we had noticed this at that time, we might have been able to arrange a check of the MR scanner earlier. The other QA metrics did not show any systematic changes before the replacement of the body coil (exemplarily shown for SNR in Fig. 3 bottom). The technical properties of the MR scanner thus remained unchanged.

### QA protocol: human MRI data

In this section, we describe the quality assessment of human MRI data (Section "Quality assessment of human MRI data"). We then show various examples of anatomical (Section "Example 1: Evaluation of anatomical MRI data") and functional data sets (Section "Example 2: Evaluation of functional MRI data") that were reduced in quality by typical artifacts and incorrect measurements.

#### Quality assessment of human MRI data

In a first step, it was checked whether the data was complete, both with regard to the MRI data and the corresponding logfiles. In particular, the correct positioning of the bounding box during the planning of the measurement was examined since a wrong alignment of the measurement volume turned out to be a frequent error source in the MRI measurements. If something was not in line with protocol, the neuroimaging centers received a direct response and additional training if necessary. However, the quality assurance protocol did not only include a check of the MRI data, but also of the entire experimental procedure. For this purpose, a clinical report form (CRF, see supplementary material S4) was designed on which the entire procedure was documented for each measurement (e.g. training of the subjects, performance of the neuropsychological tests) and unexpected events could be logged. This CRF had to be filled out for each measurement and helped to reconstruct the measurement and any problems that may have occurred.

After the initial check, the MRI data was converted into the BIDS format [using heudiconv, version v0.6.0, Halchenko et al. (2019)]. The data quality was assessed using the BIDS-App MRIQC [Magnetic Resonance Imaging Quality Control, version 0.15.2, Esteban et al. (2017)]. MRIQC assesses both structural T1-weighted MR images and blood oxygenation level dependent (BOLD)-images of the brain by calculating a set of quality measures from each image. MRIQC provides different image quality metrics (IQMs) to characterize anatomical and functional MR images. For the anatomical image, the IQMs are often divided into four broad categories. The first category comprises measures that describe the impact of noise, the second category contains metrics that characterize the spatial distribution. The measures in the third category can be used to detect artifacts. In the fourth category, all metrics are grouped that do not fit within the previous categories and characterize for instance the statistical properties of tissue distributions or the blurriness of the images. For the functional images, the IQMs are typically divided into three categories assessing spatial information, temporal information and the presence of artifacts (for an overview, see https://mriqc.readthedocs.io/).

MRIQC creates automatically visual reports for each anatomical image and each fMRI data set, respectively. Additionally, after all data was acquired, a group report was created. This report shows a plot of all individual IQMs, making it easily possible to identify outliers in each metric. These visual reports were checked by one member of the coordination team with respect to, e.g., movement, ghosting artifacts, positioning of the measurement volume, as well as the general quality of the dataset. The quality of each anatomical image and each functional time series was finally evaluated as “good”, “intermediate” or “poor” (for an overview on all data of the study, see Table 4). The label “good” was given if the rater did not see any relevant artifacts. Images that had a bit of movement and some artifacts were labeled “intermediate”. Images which had major issues (in particular strong movement, wrong placement of the measurement volume, fold-over artifacts, ghosting artifacts) were categorized as “poor”.

**Table 4:** Number of MRI data sets classified “good”, “intermediate” or “poor” based on the quality assessment with MRIQC

Dataset	“Good”	“Intermediate”	“Poor”
Structural image	171	226	50
Resting-state fMRI	305	135	6
Paradigm “DRD” (part 1/part 2)	643 (320/323)	34 (17/17)	8 (6/2)
Paradigm “Face”	330	103	8
Paradigm “ToM”	341	87	9
Total	1790	585	81

The evaluation was performed for the T1-weighted, high-resolution structural images and for each fMRI time series. Each subject was measured with four paradigms (resting-state, desire–reason dilemma (DRD) task (two sessions), emotion face matching task, Theory of Mind (ToM) task)

#### Example 1: evaluation of anatomical MRI data

**Fig. 4:**
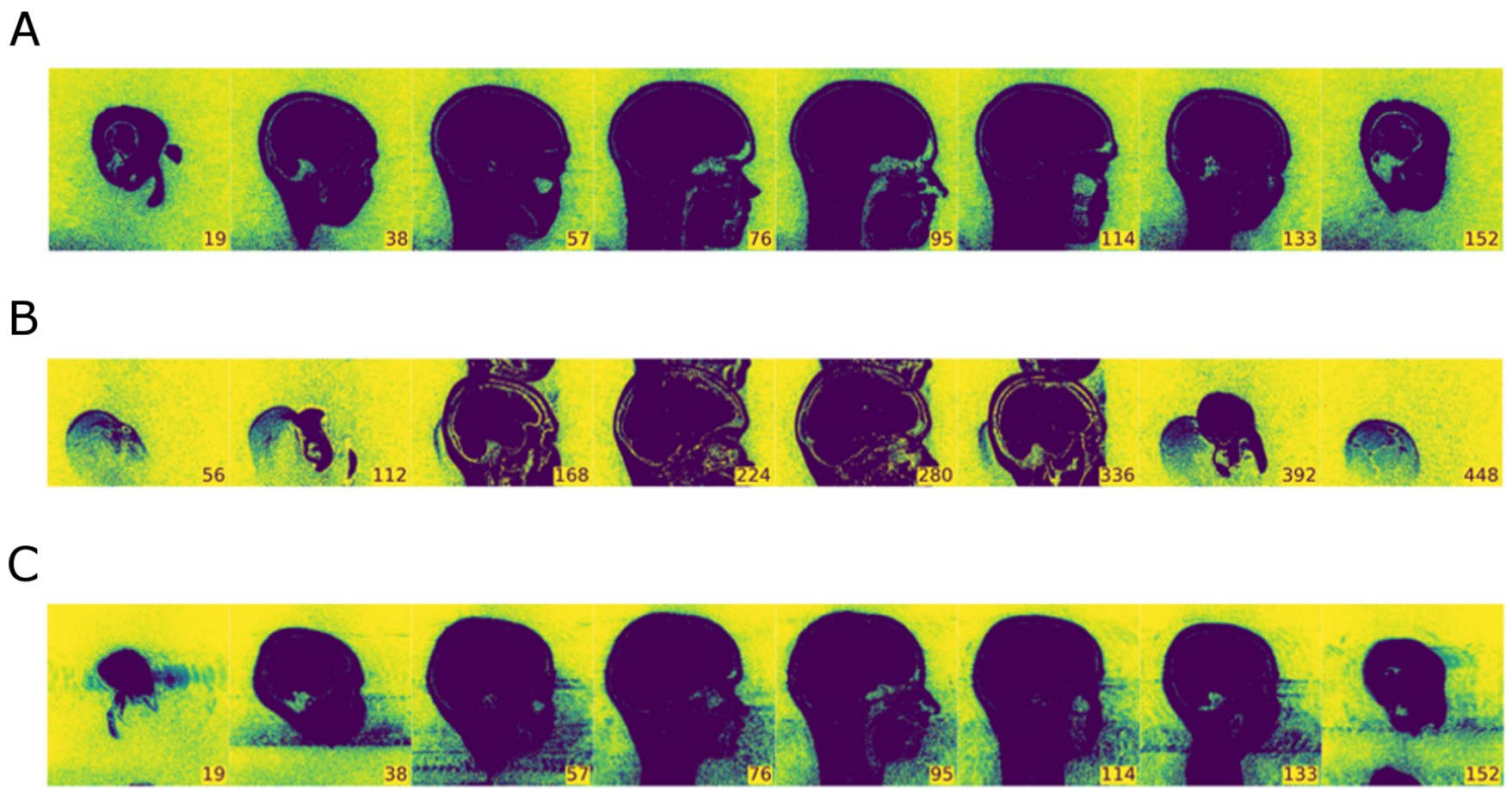
The background noise image of the structural MRIQC report for three selected subjects. The data set in **A** shows no visible artifacts and was labeled as “good”. Image **B** shows strong artifacts caused by both a bad positioning of the measurement volume (too low) a wrong phase encoding direction. This data set was labeled as “poor” and was excluded from further analyses. The data set in **C** shows strong movement artifacts extending into the prefrontal cortex and was also labeled as “poor”

In Fig. 4, we present background noise images of the MRIQC report for three selected structural MRI data sets. On top (A), we present a reference data set with no apparent artifacts. In the middle (B) and on the bottom (C), you can see images with clear artifacts that were both labeled as “poor” (for details, see figure legend).

#### Example 2: evaluation of functional MRI data

**Fig. 5:**
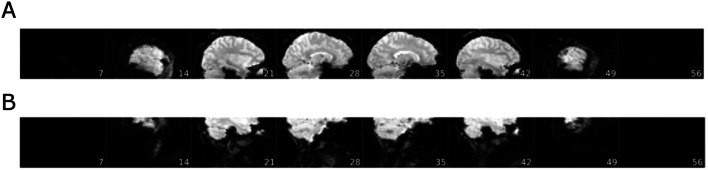
Extracts of the MRIQC report for two resting-state fMRI data sets (averaged functional image). The data set **A** shows no visible artifacts and was labeled as “good”. The image **B** indicates a wrong alignment of the measurement volume and was consequently labeled as “poor”. Please note that in data set **A**, the last slice of the cerebellum was chopped off during image acquisition. Here, the measurement volume size selected in the study protocol was too small because the subject had a relatively large brain. In these cases, we specified that the measurement volume would be positioned in such a way that the cerebrum would definitely be measured, even if parts of the cerebellum would not be measured

In Fig. 5, we present extracts of the MRIQC report (i.e., an averaged functional image) for two selected data sets acquired during the resting state paradigm. On top (A), we again present a reference data set (labeled as “good”) set with no apparent artifacts. On the bottom (B), one can clearly see that the measurement volume was wrongly aligned and did not include the whole brain. The data set was consequently labeled as “poor”. As it turned out, misalignments of the bounding box during the planning of the measurement were a major source of error in the study.

**Fig. 6:**
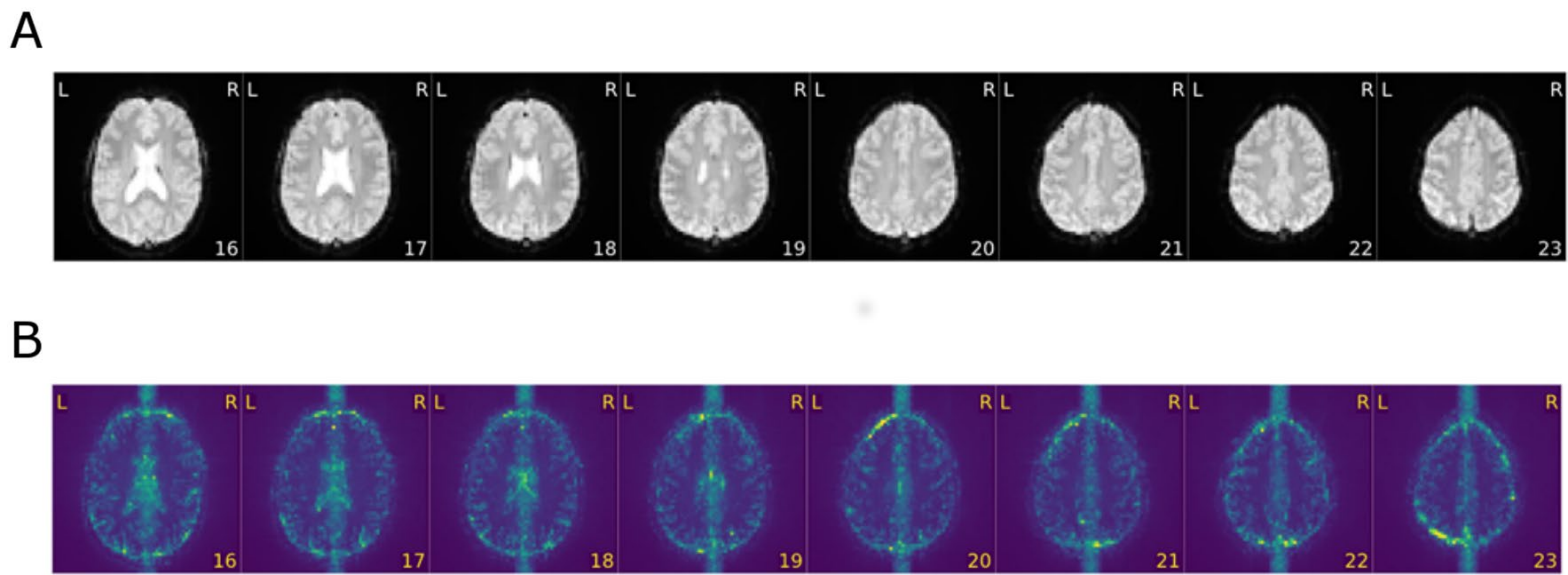
Extracts of the MRIQC report for a selected resting-state fMRI data set. Unlike the averaged functional image (**A**), the standard deviation map (**B**) clearly indicates a strong artifact

In Fig. 6, we present the averaged functional image (A) and the background noise image (B) of yet another resting-state data set. This graphic illustrates that it is important to check more than one QA metric, because some artifacts are just visible in some metrics, but not in others. More specifically, the averaged image does not show any artifacts, while the standard deviation map a clear artifact is visible.

### Influence of the quality of MRI data on brain imaging analyses

**Fig. 7:**
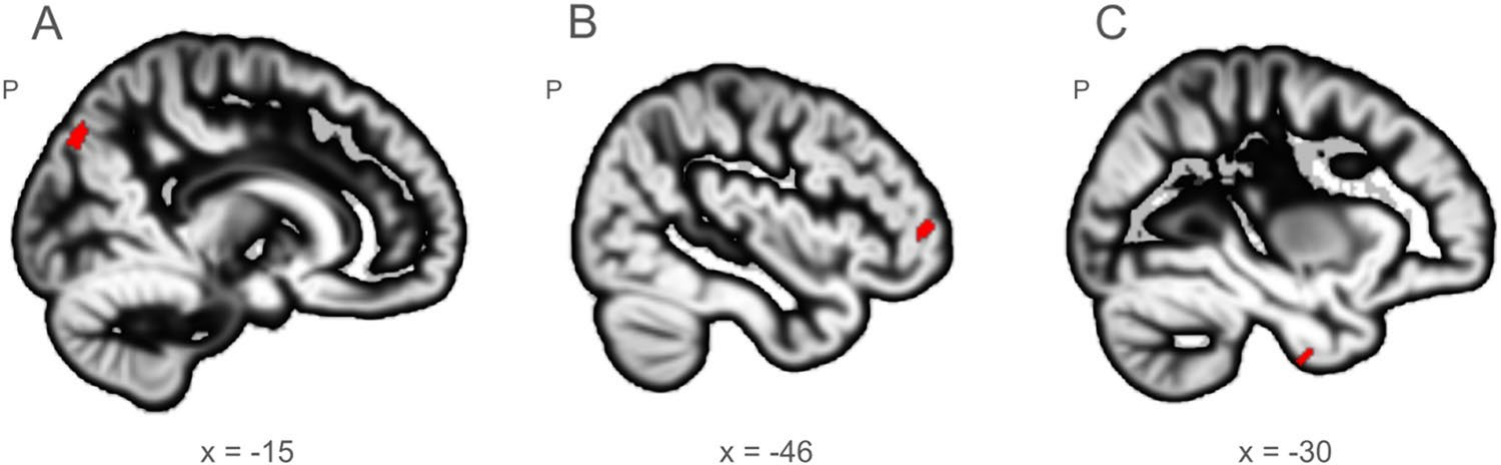
*Voxel-based analysis of anatomical MRI data:* Forty high-resolution, T1-weighted MR images of patients with major depression were drawn from the BipoLife data set. The data was preprocessed using the CAT12 Toolbox (Computational Anatomy toolbox, v1720, Structural Brain Mapping Group, Jena, Germany; https://neuro-jena.github.io/cat/), as implemented in SPM12 (Statistical Parametric Mapping, Institute of Neurology, London, UK) running on MATLAB (version R2017a, The MathWorks, Natick, Massachusetts, USA). Preprocessing steps included spatial normalization, segmentation (absolute threshold for gray matter set to 0.1) and spatial smoothing (8 mm full-width-at-half-maximum). The data set was divided into two groups (*n* = 20 each) based on the SNR values obtained from the MRIQC analysis. We did not balance for sex or site. In the high-quality group, there were 3 females and 17 males, in the low-quality group 7 females and 13 males. Gray matter segments between the high quality and the low quality group were compared between groups using a F-test with age, total-intracranial volume and sex as additional covariates. Significant differences (*p* < 0.05, family-wise error corrected for multiple comparisons at the peak level across the whole brain) were found in several brain regions including the precuneus (**A**), midfrontal gyrus (**B**) and inferior temporal gyrus (**C**). These differences can be attributed to the quality of the underlying MRI data. For visualization we used the tool MRIcroGL in version 1.2.20220720 including the DARTEL template (Rorden and Brett 2000)

The quality of the underlying data can strongly influence the results of MRI analyses (see Friedman and Glover 2006; Stöcker et al. 2005; Vogelbacher et al. 2018) for various examples from other consortia; see (Goto et al. 2016; Power et al. 2015; Zaitsev et al. 2017) for the impact of motion artifacts on fMRI data). It is not possible to assess in advance how large this influence will be for every conceivable form of analysis. The effect of data quality on the analysis results depends on many factors, e.g., the type of quality reduction, the number of subjects, the characteristics of the MR scanners involved, and, of course, the form of the specific analysis itself. Therefore, the general QA protocol, as outlined in the present article, has to be complemented by more specific QA assessments in subsequent projects. For instance, the adjustment of smoothness across MR scanners (Friedman and Glover 2006) or the introduction of specific covariates might be more important for some analyses than for others. There is not one simple solution for all such potential problems. In Fig. 7, we exemplarily illustrate that the quality of MRI data potentially can influence the results of typical neuroimaging analyses. For this purpose, we randomly selected 40 subjects from the BipoLife dataset, ordered them according to their SNR values of their T1-weighted structural image and built two groups. In the low-SNR group were those 20 subjects with the lowest values, in the high-SNR group those with the highest values. We then tested whether a standard VBM analysis showed differences between the two groups. In our example, we found significant differences in several regions (Fig. 7).

## Discussion

In this article, we provided an overview of the MRI data quality assurance protocol implemented in the BipoLife consortium. This protocol included in particular the evaluation of the MR scanner performance by regular phantom measurements and the systematic assessment of the quality of human MRI data. We also exemplified the influence of data quality on the results of a standard neuroimaging analysis pipeline for human MRI data. In the following sections, we discuss what conclusions can be drawn from the analysis of the quality of the phantom (Section "Analysis of phantom MRI data") and the human MRI data (Section "Quality assessment of human MRI data") and discuss the impact of a systematic QA protocol on a longitudinal, multicenter MRI study (Section "Impact of the introduction of a systematic QA protocol").

### Analysis of phantom MRI data

The analysis of the phantom data can be used in particular to characterize different MR scanners and to record their stability over the course of the study.

*Characterization of MR scanners*: Analyzing the phantom data using the various QA metrics makes it possible to describe the individual MR scanners according to a wide range of parameters. As expected, there were differences between the various MR scanners. These differences were scanner-specific, i.e., they also differed significantly between identical models used at different locations. How to deal with these differences in the analyses of human MRI data cannot be said in general. Of course, it is always advisable to consider the influence of the MR scanner via covariates, but it is also possible to go beyond this for certain analyses. VBM analyses of structural data, for example, require images with a good differentiation between grey and white matter, as these parameters particularly influence the segmentation of the images. Therefore, one may consider, for example, testing MR scanners with poor CNR properties to see if the data is of sufficient quality.

*Temporal stability of MR scanners*: Scanner stability is obviously key to successful MRI research, in particular for large-scale longitudinal studies. Another main application of analyzing phantom data is therefore to record the temporal stability of an MR scanner over the course of the study. On the one hand, this analysis can be used to identify outliers, i.e. points in time at which the quality characteristics of an MR scanner—for whatever reason—deviate significantly from the mean value. This information can be used to exclude specific data or at least to scrutinize the MRI data collected on these days.

On the other hand, this analysis is important in order to recognize at an early stage whether an MR scanner is beginning to deliver data of poor quality, for example because there are unrecognized technical defects or because external influences, e.g., major reconstruction work in the vicinity, are affecting data acquisition. It is possible to implement an early warning system based on data that has already been collected. The characteristic values of the last 10–20 phantom measurements can be used to calculate typical scatter ranges. If a measured value deviates from the mean value by 2.5 standard deviations, for example, it should be checked whether there is an easily recognizable reason for these deviations. Most of the deviations that occurred were not actually due to malfunction of the MR scanners, but were caused, for example, by inaccurate positioning of the phantom or incorrect selection of the sequence parameters. However, we also used an example to show that it would have been possible to recognize malfunctions in an MR scanner (caused by a deficient body coil in Marburg) at an earlier stage.

We would like to point out that in all centers except Marburg the phantom was always placed manually. In Marburg, a special phantom holder was developed. This meant that the phantom was always placed in the same position. This reduced the variance of the QA values and minimized the risk of incorrect positioning. This is of course an invaluable advantage, especially when analyzing the course of the QA values over time. We therefore recommend that similar positioning aids be used at all centers in future studies [for a thorough discussion, see Vogelbacher et al. (2018)].

### Quality assessment of human MRI data

We have demonstrated with some examples that the output of the MRIQC analysis can be used to recognize some typical problems with MRI measurements, e.g., incorrect positioning, strong movement artefacts or ghosting artefacts. Based on these metrics, we have labelled the images as "good", "intermediate" or "poor". This labelling of MRI data is of course subjective to a certain degree. Ultimately, however, our aim was not to make irrefutable quality assessments based on a universally valid criterion, but to implement a systematic screening of the MRI data by an experienced colleague prior to further analyses. This screening should provide those colleagues who coordinate the further, content-driven analyses with initial indications of which data can and cannot be included in these analyses. While it was clear for many of the data rated as "poor" that further analysis did not make sense (e.g., due to strong artefacts or incorrect positioning, see Figs. 4, 5, and 6), in other cases an individual decision had to be made based on the respective analysis. Often the analysis of the quality of fMRI data is only done based on the movement parameters, in particular for smaller studies. MRIQC, however, provides for each functional and structural dataset an individual quality report delivering a large variety of information on the acquired data. The consistent inspection of these reports allows a deeper look into the quality of human MRI datasets. We believe that the inspection of each individual report is necessary to get on the one hand a better feeling for the quality of the dataset and on the other hand to detect poor datasets.

We did not perform a formal assessment of the inter-rater reliability of our assessments. However, we had regular team meetings in which we discussed the labeling of the quality of the data, in particular for controversial cases. If we noted an artifact, this information was recorded. In retrospect, it would have been better to create a manual in advance (or at least during the ongoing process) that more systematically describes the nature of the artifacts. This would have also increased the inter-rater reliability of the quality assessments.

### Impact of the introduction of a systematic QA protocol

The question naturally arises as to what influence quality assurance has on content-motivated analyses [e.g. with regard to the question of whether one can predict the risk of developing bipolar disorder on the basis of imaging data, Huth et al. (2023); Mikolas et al. (2023); Mikolas et al. (2021)]. Ultimately, we cannot say this for all cases, as it naturally depends, among many other things, on the specific question, the analysis method and also the sample size. There is ample evidence in the literature that the quality of the MRI data has a major impact on the results of neuroimaging analyses (e.g., Friedman and Glover 2006; Haddad et al. 2019; McLaughlin et al. 2021; Stöcker et al. 2005; Sunderland et al. 2019; Vogelbacher et al. 2018). We have illustrated this effect using a standard VBM analysis on a randomly chosen subject sample.

One can—and must—ask what influence the implementation of a systematic QA protocol has on the BipoLife study. There are two main aspects to be mentioned here. The first aspect is technically motivated. With the QA protocol, we are better able to characterize the various MR scanners, record their performance over the course of the study and detect possible malfunctions at an early stage or at least classify them retrospectively. We are able to record the quality of the human MRI data systematically and to characterize the influence on various analyses. We are therefore able to take into account the influence of the MR scanner in all analyses, e.g., by using scanner-specific covariates. More importantly, the differences between the scanner models suggest that specific characteristics may differ so much that a direct quantitative comparison of two individual subjects recorded on different scanners is not readily possible. The second, and perhaps even more important, aspect goes beyond purely technical issues. The BipoLife data set was collected over several years and is continuously analyzed. By setting up and publishing this protocol, we define standards that must be observed when analyzing the data. This protocol ultimately increases the pressure to systematically consider aspects of data quality and to implement standardized analysis methods and subject selection criteria. In the long term, this increases hopefully the chance of achieving clinically relevant results.

## Supplementary Information

Supplementary Material 1.

## Data Availability

No datasets were generated or analysed during the current study.

## Abbreviations

MRI: Magnetic resonance imaging
QA: Quality assurance
BD: Bipolar disorder
fMRI: Functional MRI
EPI: Echo planar imaging
CRF: Clinical report form
MRIQC: Magnetic resonance imaging quality control
IQMs: Image quality metrics
BOLD: Blood oxygenation level dependent
GSR: Global signal regression
WM: White matter
CSF: Cerebrospinal fluid
ROIs: Regions-of-interest
SNR: Signal-to-noise ratio
CAT12-Toolbox: Computation anatomy toolbox for SPM
SPM12: Statistical parametric mapping
PSG: Percent-signal-ghosting
CNR: Contrast-to-noise ratio
DRD: Desire-reason dilemma

## References

[CR1] Bauer MS. Bipolar Disorder. Ann Intern Med. 2022;175(7):ITC97-112. 35816713 10.7326/AITC202207190

[CR2] Esteban O, Birman D, Schaer M, Koyejo OO, Poldrack RA, Gorgolewski KJ. MRIQC: advancing the automatic prediction of image quality in MRI from unseen sites. PLoS ONE. 2017;12(9):e0184661. 28945803 10.1371/journal.pone.0184661 PMC5612458

[CR3] Friedman L, Glover GH. Report on a multicenter fMRI quality assurance protocol. J Magn Reson Imaging. 2006;23(6):827–39. 16649196 10.1002/jmri.20583

[CR4] GBD 2019 Mental Disorders Collaborators. Global, regional, and national burden of 12 mental disorders in 204 countries and territories, 1990–2019: a systematic analysis for the Global Burden of Disease Study 2019. Lancet Psychiatry. 2022;9(2):137–50. 10.1016/S2215-0366(21)00395-3 PMC8776563 35026139

[CR5] Glover GH, Mueller BA, Turner JA, Van Erp TGMM, Liu TT, Greve DN, et al. Function biomedical informatics research network recommendations for prospective multicenter functional MRI studies. J Magn Reson Imaging. 2012;36(1):39–54. 22314879 10.1002/jmri.23572 PMC3349791

[CR6] Goto M, Abe O, Miyati T, Yamasue H, Gomi T, Takeda T. Head motion and correction methods in resting-state functional MRI. Magn Reson Med Sci. 2016;15(2):178–86. 26701695 10.2463/mrms.rev.2015-0060 PMC5600054

[CR7] Haddad SMH, Scott CJM, Ozzoude M, Holmes MF, Arnott SR, Nanayakkara ND, et al. Comparison of quality control methods for automated diffusion tensor imaging analysis pipelines. PLoS ONE. 2019;14(12): e0226715. 31860686 10.1371/journal.pone.0226715 PMC6924651

[CR8] Halchenko Y, Goncalves M, Castello MV di O, Ghosh S, Hanke M, Dae, et al. nipy/heudiconv v0.6.0. Zenodo; 2019. https://zenodo.org/records/3579455. Accessed 17 May 2024

[CR9] Hellerbach. Hellerbach_Dis. 2013. http://archiv.ub.uni-marburg.de/diss/z2014/0048

[CR10] Huth F, Tozzi L, Marxen M, Riedel P, Bröckel K, Martini J, et al. Machine learning prediction of estimated risk for bipolar disorders using hippocampal subfield and amygdala nuclei volumes. Brain Sci. 2023;13(6):870. 37371350 10.3390/brainsci13060870 PMC10296102

[CR11] Lu W, Dong K, Cui D, Jiao Q, Qiu J. Quality assurance of human functional magnetic resonance imaging: a literature review. Quant Imaging Med Surg. 2019;9(6):1147–62. 31367569 10.21037/qims.2019.04.18 PMC6629553

[CR12] McIntyre RS, Berk M, Brietzke E, Goldstein BI, López-Jaramillo C, Kessing LV, et al. Bipolar disorders. The Lancet. 2020;396(10265):1841–56. 10.1016/S0140-6736(20)31544-0 33278937

[CR13] McLaughlin PM, Sunderland KM, Beaton D, Binns MA, Kwan D, Levine B, et al. The quality assurance and quality control protocol for neuropsychological data collection and curation in the Ontario neurodegenerative disease research initiative (ONDRI) study. Assessment. 2021;28(5):1267–86. 32321297 10.1177/1073191120913933

[CR14] Mikolas P, Bröckel K, Vogelbacher C, Müller DK, Marxen M, Berndt C, et al. Individuals at increased risk for development of bipolar disorder display structural alterations similar to people with manifest disease. Transl Psychiatry. 2021;11(1):485. 34545071 10.1038/s41398-021-01598-y PMC8452775

[CR15] Mikolas P, Marxen M, Riedel P, Bröckel K, Martini J, Huth F, et al. Prediction of estimated risk for bipolar disorder using machine learning and structural MRI features. Psychol Med. 2023;22:1–11. 10.1017/S0033291723001319 37212052

[CR16] Pfennig A, Leopold K, Martini J, Boehme A, Lambert M, Stamm T, et al. Improving early recognition and intervention in people at increased risk for the development of bipolar disorder: study protocol of a prospective-longitudinal, naturalistic cohort study (Early-BipoLife). Int J Bipolar Disorders. 2020;8(1):22. 10.1186/s40345-020-00183-4 PMC7326843 32607662

[CR17] Power JD, Schlaggar BL, Petersen SE. Recent progress and outstanding issues in motion correction in resting state fMRI. Neuroimage. 2015;15(105):536–51. 10.1016/j.neuroimage.2014.10.044 PMC4262543 25462692

[CR18] Ritter PS, Bermpohl F, Gruber O, Hautzinger M, Jansen A, Juckel G, et al. Aims and structure of the German Research Consortium BipoLife for the study of bipolar disorder. Int J Bipolar Disorders. 2016;4(1):26. 10.1186/s40345-016-0066-0 PMC5118379 27873290

[CR19] Rorden C, Brett M. Stereotaxic display of brain lesions. Behav Neurol. 2000;12(4):191–200. 11568431 10.1155/2000/421719

[CR20] Saraf G, Moazen-Zadeh E, Pinto JV, Ziafat K, Torres IJ, Kesavan M, et al. Early intervention for people at high risk of developing bipolar disorder: a systematic review of clinical trials. Lancet Psychiatry. 2021;8(1):64–75. 32857954 10.1016/S2215-0366(20)30188-7

[CR21] Sreedher G, Ho M-L, Smith M, Udayasankar UK, Risacher S, Rapalino O, et al. Magnetic resonance imaging quality control, quality assurance and quality improvement. Pediatr Radiol. 2021;51(5):698–708. 33772641 10.1007/s00247-021-05043-6

[CR22] Stöcker T, Schneider F, Klein M, Habel U, Kellermann T, Zilles K, et al. Automated quality assurance routines for fMRI data applied to a multicenter study. Hum Brain Mapp. 2005;25:237–46. 15846770 10.1002/hbm.20096 PMC6871722

[CR23] Sunderland KM, Beaton D, Fraser J, Kwan D, McLaughlin PM, Montero-Odasso M, et al. The utility of multivariate outlier detection techniques for data quality evaluation in large studies: an application within the ONDRI project. BMC Med Res Methodol. 2019;19(1):102. 31092212 10.1186/s12874-019-0737-5 PMC6521365

[CR24] Van Horn JD, Toga AW. Multisite neuroimaging trials. Curr Opin Neurol. 2009;22(4):370–8. 19506479 10.1097/WCO.0b013e32832d92de PMC2777976

[CR25] Vogelbacher C, Möbius TWD, Sommer J, Schuster V, Dannlowski U, Kircher T, et al. The Marburg-Münster affective disorders Cohort study (MACS): a quality assurance protocol for MR neuroimaging data. Neuroimage. 2018;172:450–60. 29410079 10.1016/j.neuroimage.2018.01.079

[CR26] Vogelbacher C, Bopp MHA, Schuster V, Herholz P, Jansen A, Sommer J. LAB–QA2GO: a free, easy-to-use toolbox for the quality assessment of magnetic resonance imaging data. Front Neurosci. 2019;13:688. 31333406 10.3389/fnins.2019.00688 PMC6617644

[CR27] Vogelbacher C, Sommer J, Schuster V, Bopp MHA, Falkenberg I, Ritter PS, et al. The German research consortium for the study of bipolar disorder (BipoLife): a magnetic resonance imaging study protocol. Int J Bipolar Disorders. 2021;9(1):37. 10.1186/s40345-021-00240-6 PMC8595454 34786613

[CR28] Vogelbacher C. Development of quality standards for multi-center, longitudinal magnetic resonance imaging studies in clinical neuroscience. University of Marburg. 2020. https://archiv.ub.uni-marburg.de/diss/z2020/0151

[CR29] Zaitsev M, Akin B, LeVan P, Knowles BR. Prospective motion correction in functional MRI. Neuroimage. 2017;1(154):33–42. 10.1016/j.neuroimage.2016.11.014 PMC5427003 27845256

