# Supplementary material for: The German research consortium for the study of bipolar disorder (BipoLife): a quality assurance protocol for MR neuroimaging data
Source: Int J Bipolar Disord. 2024 Sep 26;12:33. doi: 10.1186/s40345-024-00354-7 (PMC11427632; doi:10.1186/s40345-024-00354-7)
Supplement: Supplementary file 1 — Supplementary Material 1. [file 40345_2024_354_MOESM1_ESM.pdf]

Supplement Material for

The German research consortium

for the study of bipolar disorder (BipoLife):

A quality assurance protocol for MR neuroimaging data

S1: Illustrating the alignment of the phantom

A: The alignment of the phantom either manually (top) or using a phantom holder (bottom)

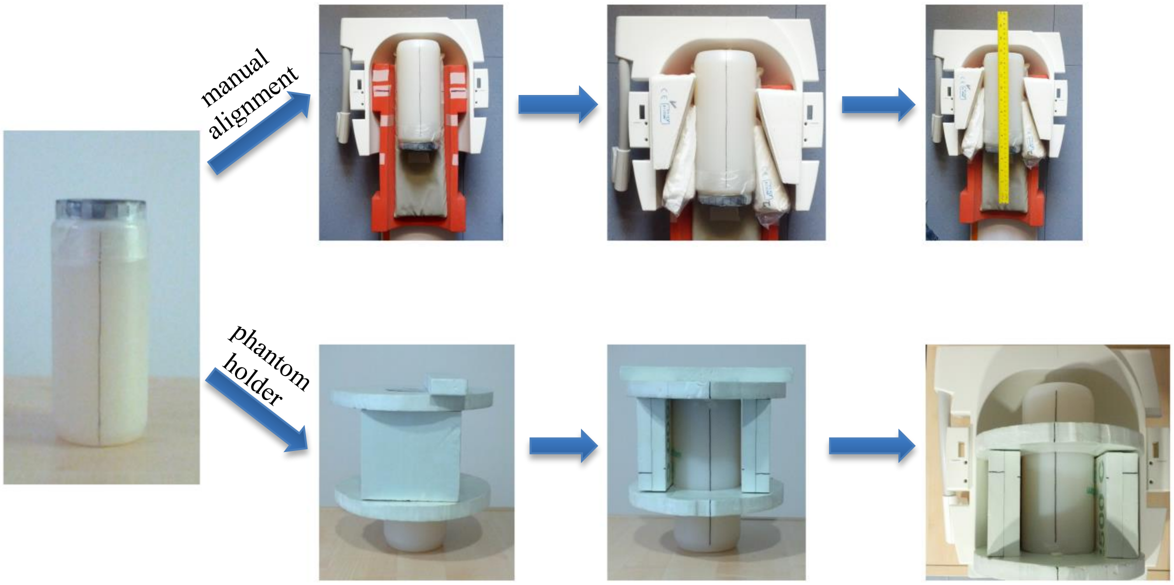

B: Alignment of the bounding box for the measurement in sagittal (left), coronal (middle) and axial view (right).

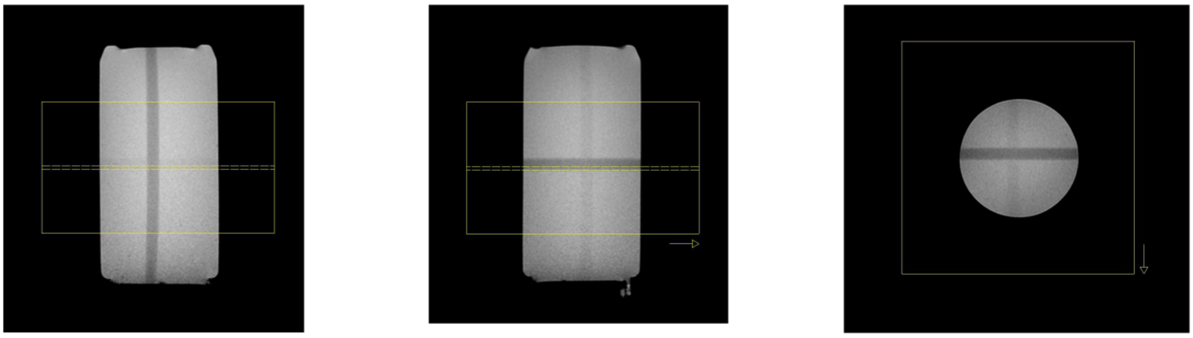

S2: Descriptive statistics of all LAB-QA2GO-based measurements for each site (minimum [min], maximum [max], mean and standard deviation [sd])

Berlin

**value**	**mean**	**min**	**max**	**sd**
SNR	164.98	36.77	209.41	20.98
SFNR	161.40	33.93	181.38	18.49
percent fluctuation	0.10	0.06	0.28	0.05
drift	1.95	1.02	3.09	0.43
PSC	1.05	0.94	3.20	0.21
PSG	0.0009	0.0	0.0027	0.0007

Bochum

**value**	**mean**	**min**	**max**	**sd**
SNR	137.53	26.82	182.79	43.32
SFNR	124.00	25.85	156.83	38.40
percent fluctuation	0.33	0.19	0.57	0.12
drift	1.39	0.19	2.63	0.82
PSC	0.058	0.026	0.13	0.03
PSG	0.0012	0.0001	0.003	0.001

Dresden

**value**	**mean**	**min**	**max**	**sd**
SNR	163.75	147.37	191.67	11.14
SFNR	162.07	131.58	180.19	8.46
percent fluctuation	0.08	0.06	0.33	0.04
drift	0.62	0.02	1.17	0.25
PSC	0.93	0.87	1.11	0.05
PSG	0.0008	0.0002	0.0015	0.0003

Frankfurt

value	mean	min	max	sd
SNR	208.31	168.82	243.03	16.01
SFNR	165.32	104.13	219.59	21.14
percent fluctuation	0.37	0.07	0.85	0.14
drift	2.08	0.50	3.01	0.53
PSC	0.6	0.55	0.66	0.02
PSG	0.0039	0.0006	0.0088	0.0012

Göttingen

value	mean	min	max	sd
SNR	163.88	132.61	177.02	14.48
SFNR	160.14	147.01	170.91	9.15
percent fluctuation	0.14	0.07	0.30	0.08
drift	1.98	1.25	3.04	0.52
PSC	0.94	0.89	1.00	0.05
PSG	0.0068	0.0021	0.0152	0.0049

Hamburg

value	mean	min	max	sd
SNR	278.23	207.10	344.19	30.86
SFNR	266.77	213.05	322.33	23.36
percent fluctuation	0.09	0.04	0.86	0.15
drift	3.56	1.19	6.01	0.96
PSC	0.74	0.38	1.16	0.21
PSG	0.0091	0.0002	0.0206	0.0049

Marburg

**value**	**mean**	**min**	**max**	**sd**
SNR	157.20	141.37	189.1	9.41
SFNR	157.88	145.97	180.996	5.66
percent fluctuation	0.09	0.06	0.19	0.03
drift	1.16	0.68	1.67	0.21
PSC	1.00	0.91	1.05	0.02
PSG	0.0021	0.004	0.0109	0.0021

Tübingen

**value**	**mean**	**min**	**max**	**sd**
SNR	231.70	135.47	362.47	52.54
SFNR	227.65	116.13	362.53	56.39
percent fluctuation	0.15	0.03	1.51	0.244
drift	0.16	-0.52	3.27	0.63
PSC	0.41	0.28	0.64	0.07
PSG	0.0094	0.0020	0.0722	0.0100

S3: Changed placement of the phantom in the MR scanner

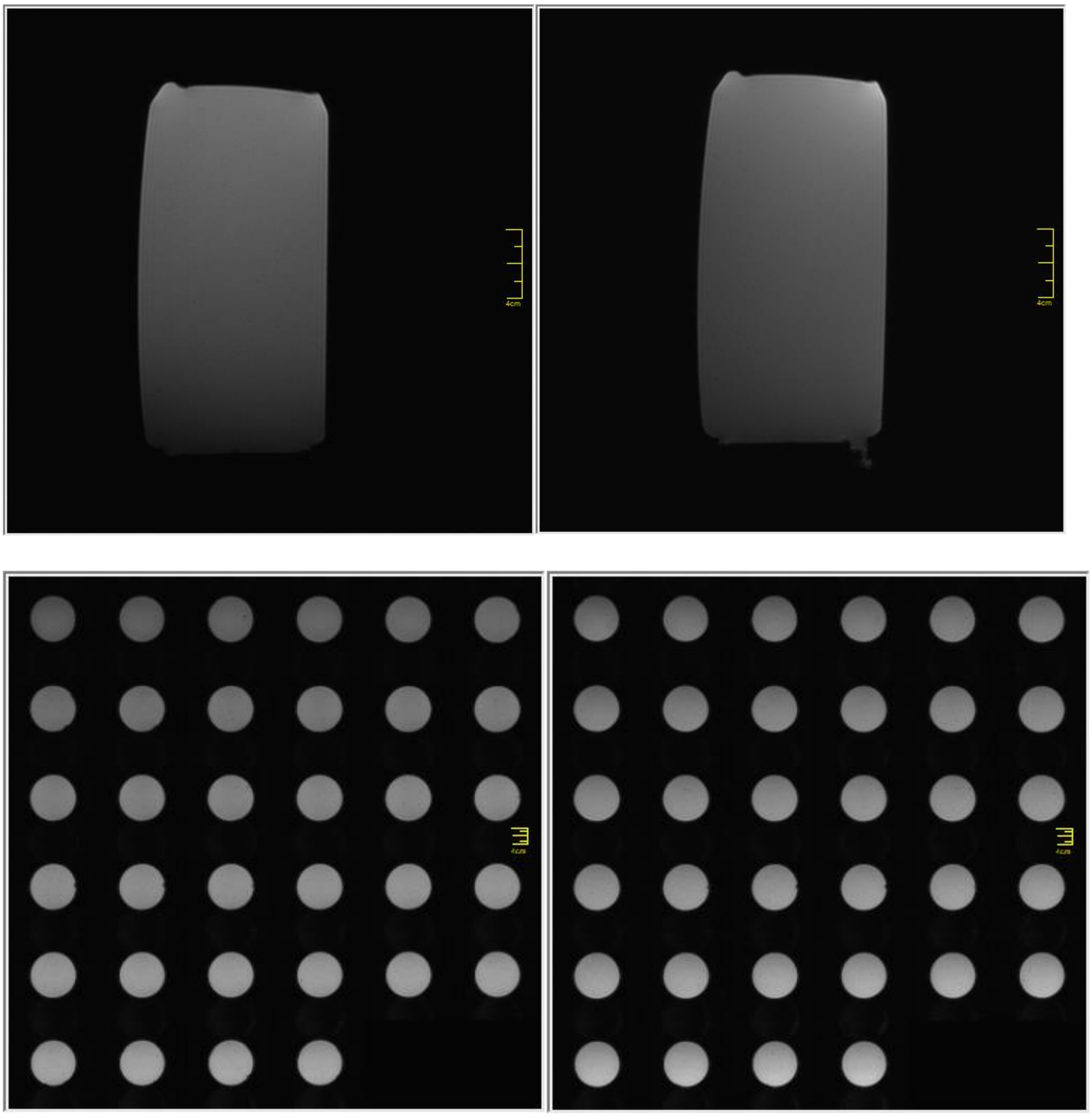

Figure S3: An analysis of the scanner drift showed at a specific measurement point significantly altered values. A closer inspection showed that these alterations were solely related to a slightly different positioning of the phantom. On the left side, we show the "outlier", on the right side a typical measurement (top: phantom in sagittal view, bottom: data acquisition slice by slice). The misplacement becomes evident when looking at the small air bubble at the right corner of the phantom (fourth row).

S4: Clinical report form (CRF)

Clinical Report Form

BIPOLIFE

Imaging A1 ☐ **Gruppe 1 (Früherkennung)** **A2** ☐ **Proband** **B2** ☐
☐ **Gruppe 2 (Depressive)** ☐ **Kontrolle**
☐ **Gruppe 3 (ADHS)**

Dieser “Laufzettel” soll für jede MR-Messung ausgefüllt werden und – zusammen mit den MRT-Daten – nach Marburg verschickt werden. Der angehängte EEG Teil sollte nach einer EEG Messung ebenfalls abgefragt werden. Bekommt ein Proband/Patient eine EEG und MR Messung kann ein gemeinsamer Laufzettel genutzt werden.

Standort:

Berlin	Bochum	Dresden	Frankfurt	Göttingen	Hamburg	Heidelberg	Marburg	Tübingen

PROBANDENINFORMATIONEN

Probandencode: ____________ **_MRT_** ____________

Datum der Messung		Beginn der Messung	
Messung 1 / Messung 2?	Messung 1 | Messung 2	Ende der Messung	
Geburtsdatum	01.01.	Geschlecht	männlich | weiblich
Datum der Testungen		MADRS (nur B2)*	
Bildungsgrad*		Händigkeitpunkte*<br>*(Links | rechts)*	L: |R:
Zahlensymboltest*<br>(in 90 Sek | davon Richtige)	90 Sek: |Richtige:	Zahlen nachsprechen*<br>(Summe)	
YMRS Gesamtscore*		IDS-C Gesamtscore*	
DRD Punkte Teil 1<br>Gesamtpunkte| davon Bonus	|	DRD Punkte Teil 2<br>Gesamtpunkte| davon Bonus	|

* werden über Bögen, die weiter hinten aufgeführt sind, bestimmt. Diese Tests vor dem Scan durchführen

**PROTOKOLLIERUNG MESSABLAUF<br>Außerhalb des Scanners**	**Bitte abhaken**	**PROTOKOLLIERUNG MESSABLAUF<br>Innerhalb des Scanners**	**Bitte abhaken**
Metallanamnese		Localizer oder AAH Scout	
Aufklärung, Einverständnis		T1	
MRT-Probandencode<br>(*Probandencode_MRT_Datum*)		Resting State	
		DRD-Aufgabe Teil 1	
Testung durchgeführt		DRD-Aufgabe Teil 2	
Übung DRD		Gesichter-Aufgabe	
Übung Gesichter		Comic-Aufgabe	
Übung Comic		Fieldmap	
**Datentransfer**<br>(Kopie des CRF, Logfiles, MRT-Daten)		T2 (nur Dresden)	

Phantommessung

Phantomcode: _FUN_

**PROTOKOLLIERUNG MESSABLAUF Außerhalb des Scanners**	**Bitte Eintragen**	**PROTOKOLLIERUNG MESSABLAUF Innerhalb des Scanners**	**Bitte abhaken**
Heliumstand*	%	Localizer	
Temperatur im Scannerraum*	°C	Funktionelle Messung	
Luftfeuchtigkeit (falls vorhanden)			

*Diese Werte werden vor dem Scan des Phantoms erfasst.

Kommentare, Besonderheiten während der Messung

(aktuelle und eingenommene) Medikation zum Zeitpunkt der Messung (Schema)

Nach der Messung findet mit dem Probanden eine kurze Nachbesprechung statt. Wichtig ist:

Konnten Sie mit Ihrer Aufmerksamkeit über die ganze Zeit gut bei den Aufgaben bleiben?

ja	nein

Wenn **nein**, bei welchen Aufgaben konnten Sie nicht mit Ihrer Aufmerksamkeit bei der Aufgabe bleiben?

FRAGEBOGEN 1: BILDUNGSGRAD

	Keine Angabe
1	Schulabgang ohne Abschluss
2	Hauptschulabschluss/ Volksschulabschluss
3	Realschulabschluss/ Mittlere Reife/ Fachschulreife
4	POS 10. Klasse
5	Fachhochschulreife/ fachgebundene Hochschulreife/ Fachoberschule
6	Allgemeine Hochschulreife (Abitur, EOS, Berufsausbildung mit Abitur)
7	Anderer Schulabschluss:
8	Noch in der Schulausbildung; angestrebter Schulabschluss:

FRAGEBOGEN 2: HÄNDIGKEIT

Halten Sie sich für [ ] rechtshändig [ ] linkshändig [ ] beidhändig

◙ *Bei den unten stehenden Fragen gilt folgende Codierungsregel:*

ausschließlich links	[x] [x]	[ ] [ ]
vornehmlich links	[ ] [x]	[ ] [ ]
links und rechts	[ ] [x]	[x] [ ]
vornehmlich rechts	[ ] [ ]	[x] [ ]
ausschließlich rechts	[ ] [ ]	[x] [x]

	links	rechts
1. Mit welcher Hand schreiben Sie einen Brief?	[ ] [ ]	[ ] [ ]
2. Mit welcher Hand malen Sie?	[ ] [ ]	[ ] [ ]
3. Mit welcher Hand werfen Sie einen Ball?	[ ] [ ]	[ ] [ ]
4. Mit welcher Hand halten Sie eine Schere beim Schneiden?	[ ] [ ]	[ ] [ ]
5. Mit welcher Hand halten Sie die Zahnbürste beim Zähneputzen?	[ ] [ ]	[ ] [ ]
6. Mit welcher Hand halten Sie ein Streichholz beim Anzünden?	[ ] [ ]	[ ] [ ]
7. Mit welcher Hand halten Sie ein Brotmesser?	[ ] [ ]	[ ] [ ]
8. Mit welcher Hand halten Sie beim Kehren das obere Ende des Besenstiels?	[ ] [ ]	[ ] [ ]
9. Mit welcher Hand halten Sie einen Löffel beim Essen?	[ ] [ ]	[ ] [ ]
10. Mit welcher Hand schrauben Sie den Deckel einer Dose auf?	[ ] [ ]	[ ] [ ]
**Summe links / rechts**		

FRAGEBOGEN 3: ZAHLENSYMBOLTEST

Anweisung für den Versuchsleiter:

Der Proband darf sich bei diesem Test korrigieren, jedoch kein Radiergummi benutzen.

Der Versuchsleiter legt den Arbeitsbogen vor den Probanden und zeigt auf den Schlüssel und sagt:

„Sehen Sie bitte diese abgeteilten Kästchen oder Vierecke an! Beachten Sie, dass jedes im oberen Teil eine Ziffer enthält und im unteren ein kleines Zeichen. Zu jeder Ziffer gehört also ein bestimmtes Zeichen".

Der Versuchsleiter weist auf das Beispiel.

„Bitte setzten Sie in jedes dieser Vierecke (zeigen) das kleine Zeichen ein, das dazu gehören würde zum Beispiel hier bei der 2 setzen wir dieses Zeichen ein (zeigen) und bei der 1 dieses (zeigen). Bitte füllen Sie jetzt die Beispiele bis zu dem fett gedruckten Strich aus".
„Nun fangen Sie nach dem fett gedruckten Strich an (zeigen) und füllen der Reihe nach, ohne eines auszulassen, so viele Vierecke aus, wie Sie können, bis ich halt sage."

Nach 90 Sekunden sagt der Versuchsleiter: *„Halt, bitte aufhören!"*

ZAHLEN – SYMBOL – TEST [HAWIE-R]

1	2	3	4	5	6	7	8	9
–	⊥	⊐	∟	⊔	○	∧	X	=

Punkte

90´´

Beispiel

2	1	3	7	2	4	8	2	1	3	2	1	4	2	3	5	2	3	1	4	5	6	3	1	4

1	5	4	2	7	6	3	5	7	2	8	5	4	6	3	7	2	8	1	9	5	8	4	7	3

6	2	5	1	9	2	8	3	7	4	6	5	9	4	8	3	7	2	6	1	5	4	6	3	7

9	2	8	1	7	9	4	6	8	5	9	7	1	8	5	2	9	4	8	6	3	7	9	8	6

Summe der Richtigen:

FRAGEBOGEN 4: Zahlennachsprechen „rückwärts“

<u>Anweisung für den Versuchsleiter:</u>

Der Versuchsleiter gibt folgende Instruktionen:

„Ich werde Ihnen jetzt einige Zahlen vorsprechen. Hören Sie bitte aufmerksam zu, weil ich Ihnen die Zahlen nur einmal sagen kann. Wenn ich fertig bin, wiederholen Sie bitte die Zahlen – aber rückwärts. Wenn ich z.B. 7-1 sage, was würden Sie dann antworten?“

FÜR INSTRUKTION
7-1 → richtige Antwort: 1-7
3-4 → richtige Antwort: 4-3

Der Test wird abgebrochen, wenn beide Versuche bei einer Aufgabe (Zahlenpaare mit gleicher Anzahl zu wiederholender Zahlen) nicht oder falsch gelöst werden.

**Aufgabe**	**Versuch**	**Richtige Antwort**	**Antwort des Probanden**	**Punkte/ Versuch**	**Erreichte Punkte**
1	3—1	1—3		0 1	0 1 2
	2—4	4—2		0 1	
2	4—6	6—4		0 1	0 1 2
	5—7	7—5		0 1	
3	6—2—9	9—2—6		0 1	0 1 2
	4—7—5	5—7—4		0 1	
4	8—2—7—9	9—7—2—8		0 1	0 1 2
	4—9—6—8	8—6—9—4		0 1	
5	6—5—8—4—3	3—4—8—5—6		0 1	0 1 2
	1—5—4—8—6	6—8—4—5—1		0 1	
6	5—3—7—4—1—8	8—1—4—7—3—5		0 1	0 1 2
	7—2—4—8—5—6	6—5—8—4—2—7		0 1	
7	8—1—4—9—3—6—2	2—6—3—9—4—1—8		0 1	0 1 2
	4—7—3—9—6—2—8	8—2—6—9—3—7—4		0 1	
8	9—4—3—7—6—2—1—8	8—1—2—6—7—3—4—9		0 1	0 1 2
	7—2—8—1—5—6—4—3	3—4—6—5—1—8—2—7		0 1	
				**Summe**	

FRAGEBOGEN 5: Fremdbeurteilungsbogen YMRS

Bitte jeweils nur die zutreffende Ziffer in die Kästchen eintragen! Bitte alle Feststellungen beantworten! Beurteile für den Zeitraum der letzten 8 Tage!

Richtlinien zur Verwendung der Skala:
Der Zweck jedes Items ist es, den Schweregrad der Symptomatik des Patienten einzuschätzen. **Es muss nur ein Kennzeichen für eine bestimmte Schwereabstufung vorhanden sind, damit diese Kodierung genommen wird!**
Die zur Verfügung stehenden Kennzeichen sind Richtlinien.
Bei hinreichender Erfahrung im Umgang mit der Skala, ist es möglich, auch zwischen den vorgegebenen Ankerpunkten zu kodieren, d.h. halbe Punkte zu vergeben. Dies ist besonders nützlich, wenn bei einem Patienten der Schweregrad eines bestimmten Items nicht der in den Kennzeichnungen angegebenen Staffelung folgt.

1. Gehobene Stimmung

☐

0 Keine
1 Leicht oder möglicherweise erhöht, wenn nachgefragt
2 Definitiv subjektiv gehoben; optimistisch, selbstbewusst; fröhlich, dem Zusammenhang angemessen
3 Gehoben, dem Zusammenhang unangemessen humorvoll
4 Euphorisch; unangebrachtes Lachen, Singen

2. Erhöhte motorische Aktivität – Energie

☐

0 Keine
1 Subjektiv erhöht
2 Angeregt; vermehrte Gestik
3 Übermäßige Energie, zeitweise hyperaktiv, unruhig (kann beruhigt werden)
4 Motorische Erregung, andauernde Hyperaktivität (kann nicht beruhigt werden)

3. Sexuelles Interesse

☐

0 Normal; nicht erhöht
1 Leicht oder möglicherweise erhöht
2 Definitive subjektive Zunahme auf Nachfrage
3 Spontan sexuell Themen ansprechend; führt sexuelle Themen aus; nach Selbstaussage hypersexuell
4 Offene sexuelle Handlungen (gegenüber Patienten, Personal oder Interviewer)

4. Schlaf

☐

0 Berichtet keinen Rückgang der Schlafdauer
1 Schläft bis zu einer Stunde weniger als normal
2 Schläft mehr als eine Stunde weniger als normal
3 Berichtet geringeres Schlafbedürfnis
4 Bestreitet Schlafbedürfnis

5. Reizbarkeit

☐

0 Keine
2 Subjektiv erhöht
4 Zeitweise reizbar während des Interviews; kürzlich Episoden von Ärger und Verdruss auf der Station
6 Häufig reizbar während des Interviews; durchgehend kurz und knapp
8 Feindselig, unkooperativ; Interview unmöglich

6. Sprechen (Tempo und Quantität)

0 Keine Zunahme
2 Fühlt sich gesprächig
4 Zeitweise erhöhtes Tempo und Quantität, zeitweise weitschweifig
6 Getrieben; ständig erhöhtes Tempo und Quantität; schwer zu unterbrechen
8 Rededrang; nicht zu unterbrechen; ständiges Reden

7. Sprache - Gedanken – Störung

0 Keine
1 Umständlich; leichte Ablenkbarkeit; schnelle Gedanken
2 Ablenkbar; verliert das Ziel aus den Augen; wechselt oft die Themen; Gedankenrasen
3 Ideenflucht; tangentiell; schwer zu folgen; Reimen, Echolalie
4 Unzusammenhängend; Kommunikation unmöglich

8. Inhalt

0 Normal
2 fragwürdige Pläne, verfolgt mehrere Dinge
4 besondere Projekte, hyperreligiös
6 Grandiose und paranoide Ideen, Beziehungsideen
8 Wahnvorstellungen; Halluzinationen

9. Störend - aggressives Verhalten

0 Keines, kooperativ
2 Sarkastisch, zeitweise laut, vorsichtig
4 Anspruchsvoll, bedroht andere
6 Bedroht den Interviewer, Schreien, Interview ist schwierig
8 Greift tätlich an, zerstörerisch, Interview unmöglich

10. Erscheinung

0 Angemessene Kleidung und gepflegtes Äußeres
1 Minimal ungepflegt
2 Ungepflegtes Äußeres, etwas unordentlich, overdressed
3 Unordentlich, teilweise bekleidet, grelles Make-up
4 Völlig ungepflegt; dekoriert, bizarre Kluft

11. Einsicht

0 Vorhanden; gibt die Krankheit zu; stimmt der Notwendigkeit zur Behandlung zu
1 Möglicherweise krank
2 Gibt Verhaltensänderung zu, aber streitet die Krankheit ab
3 Gibt mögliche Verhaltensänderung zu, aber streitet die Krankheit ab
4 Streitet jegliche Verhaltensänderung ab

Gesamtscore :

FRAGEBOGEN 6: Liste Depressiver Symptome (Fremdbeurteilung) (IDS-C)

Bitte kreuzen Sie zu jedem der folgenden Symptome eine Antwort an, die den Patienten während der vergangenen 7 Tage am besten beschreibt.

1. Einschlafschwierigkeiten:

0 Patient brauchte nie länger als 30 Minuten um einzuschlafen.
1 Patient brauchte an weniger als der Hälfte der vergangenen 7 Tage mindestens 30 Minuten um einzuschlafen.
2 Patient brauchte an mehr als der Hälfte der 7 Tage mindestens 30 Minuten um einzuschlafen.
3 Patient brauchte an mehr als der Hälfte der 7 Tage mehr als 60 Minuten um einzuschlafen.

☐

2. Nächtliches Aufwachen:

0 Patient wachte während der Nacht nicht auf.
1 Patient hatte einen unruhigen, leichten Schlaf und wachte einige Male auf.
2 Patient wachte mindestens einmal pro Nacht auf, schlief jedoch leicht wieder ein.
3 Patient wachte an mehr als der Hälfte der 7 Tage mehr als einmal pro Nacht auf und blieb länger als 20 Minuten wach.

☐

3. Frühes Aufwachen:

0 Patient wachte an weniger als der Hälfte der vergangenen 7 Tage weniger als eine halbe Stunde früher auf als notwendig.
1 Patient wachte an mehr als der Hälfte der 7 Tage mehr als eine halbe Stunde früher auf als notwendig.
2 Patient wachte an mehr als der Hälfte der 7 Tage eine Stunde früher auf als notwendig.
3 Patient wachte an mehr als der Hälfte der 7 Tage zwei Stunden früher auf als notwendig.

☐

4. Hypersomnie:

0 Patient schlief weniger als 7-8 Stunden (ohne Nickerchen).
1 Patient schlief innerhalb von 24 Stunden weniger als 10 Stunden (einschl. Nickerchen).
2 Patient schlief innerhalb von 24 Stunden weniger als 12 Stunden (einschl. Nickerchen).
3 Patient schlief innerhalb von 24 Stunden mehr als 12 Stunden (einschl. Nickerchen).

☐

5. Stimmung (Traurigkeit):

0 Patient fühlte sich nicht traurig.
1 Patient fühlte sich an weniger als der Hälfte der letzten 7 Tage traurig.
2 Patient fühlte sich an mehr als der Hälfte der letzten 7 Tage traurig.
3 Patient fühlte sich praktisch die gesamten letzten 7 Tage über sehr traurig.

☐

6. Stimmung (Gereiztheit):

0 Patient fühlte sich nicht gereizt.
1 Patient fühlte sich an weniger als der Hälfte der letzten 7 Tage gereizt.
2 Patient fühlte sich an mehr als der Hälfte der letzten 7 Tage gereizt.
3 Patient fühlte sich praktisch an allen 7 Tagen äußerst gereizt.

☐

7. Stimmung (Angst):

0 Patient fühlte sich nicht ängstlich oder angespannt.
1 Patient fühlte sich an weniger als der Hälfte der letzten 7 Tage ängstlich oder angespannt.
2 Patient fühlte sich an mehr als der Hälfte der letzten 7 Tage ängstlich/angespannt.
3 Patient fühlte sich praktisch die ganze Zeit äußerst ängstlich/angespannt.

☐

8. Reaktivität der Stimmung:

0 Nach einem positiven Ereignis verbesserte sich die Stimmung des Patienten bis auf das normale Niveau und hielt mehrere Stunden an.
1 Nach einem positivem Ereignis verbesserte sich die Stimmung des Patienten zwar, doch das normale Niveau wurde nicht erreicht.
2 Patient zeigte nach wenigen und von sich sehr erwünschten Ereignissen nur eine geringe S timmungsverbesserung.
3 Patient zeigte selbst nach sehr positiven oder erwünschten Ereignissen keine Stimmungsverbesserung.

9. Stimmungsschwankungen:

0 Zwischen der Stimmung des Patienten und der Tageszeit war kein offensichtlicher Zusammenhang festzustellen.
1 Die Stimmung des Patienten stand aufgrund von äusseren Umständen mit bestimmten Tageszeiten im Zusammenhang.
2 Meistens schien die Stimmung des Patienten mehr von der Tageszeit als von Ereignissen abhängig zu sein.
3 Die Stimmung des Patienten war zu einer bestimmten Tageszeit eindeutig und vorhersagbar besser oder schlechter.

9A. Wann war die Stimmung normalerweise schlechter? **(Bitte eine Antwort Ankreuzen)**.

□ Morgens
□ Nachmittags
□ Abends

9B. Wurde die Stimmung des Patienten von seiner Umgebung beeinflusst? **(Bitte eine Antwort Ankreuzen)**.

□ Ja
□ Nein

10. Qualität der Stimmung:

0 Die Stimmung des Patienten war nicht gestört oder entsprach weitgehend Gefühlen, die bei Verlusten auftreten.
1 Die Stimmung des Patienten ähnelte meist der Traurigkeit bei Verlust einer nahestehenden Person, obgleich sie nicht immer zu erklären und mit mehr Angst verbunden war, oder sehr viel intensiver sein konnte.
2 Die Traurigkeit des Patienten unterschied sich an weniger als der Hälfte der vergangenen 7 Tage qualitativ deutlich von der Trauer bei Verlust einer nahestehenden und war daher anderen schwer zu erklären.
3 Die Stimmung des Patienten unterschied sich fast die ganze Zeit qualitativ von einer Trauer bei Verlust einer nahestehenden Person.

BITTE ENTWEDER 11 <u>ODER</u> 12 BEANTWORTEN!

11. Appetit (Verminderung):

0 Patient zeigte keine Veränderung des gewöhnlichen Appetits.
1 Patient aß etwas weniger oft und/oder geringere Mengen als gewöhnlich.
2 Patient aß deutlich weniger als gewöhnlich und nur unter Anstrengung.
3 Patient aß selten im Verlauf von 24 Stunden und nur mit größter Anstrengung oder nach Überredung durch andere.

12. Appetit (Steigerung):

0 Patient zeigte keine Veränderung des gewöhnlichen Appetits.
1 Patient verspürte häufig das Bedürfnis mehr als üblich zu essen.
2 Patient aß regelmäßig mehr als gewöhnlich und/oder größere Mengen.
3 Patient fühlte den Drang zuviel zu essen, sowohl bei den Mahlzeiten als auch zwischendurch.

BITTE ENTWEDER 13 ODER 14 BEANTWORTEN!

13. Gewichtsabnahme (während der letzten 2 Wochen):

0 Patient nahm nicht an Gewicht ab.
1 Patient fühlte, dass er etwas abgenommen hat.
2 Patient nahm 1-2 kg ab.
3 Patient nahm 2 kg oder mehr ab.

14. Gewichtszunahme (während der letzten 2 Wochen):

0 Patient nahm nicht an Gewicht zu.
1 Patient nahm etwas zu.
2 Patient nahm 1-2 kg zu.
3 Patient nahm 2 kg oder mehr zu.

15. Konzentration, Entscheidungsvermögen:

0 Konzentrations- und Entscheidungsvermögen des Patienten war unverändert.
1 Patient fühlte sich gelegentlich unentschlossen und unaufmerksam.
2 Patient hatte die meiste Zeit Schwierigkeiten, sich zu konzentrieren oder sich zu entscheiden.
3 Patient konnte sich nicht ausreichend auf das Lesen konzentrieren oder selbst kleine Entscheidungen nicht treffen.

16. Selbstbild:

0 Patient betrachtete sich als ebenso wertvoll und verdienstvoll wie andere Menschen.
1 Patient machte sich öfter als gewöhnlich Vorwürfe.
2 Patient glaubte, dass er/sie für andere nur eine Last sei und Probleme verursache.
3 Patient grübelte über viele seiner größeren und kleineren Fehler.

17. Sicht der Zukunft:

0 Patient sah die Zukunft mit üblichem Optimismus.
1 Patient hatte gelegentlich pessimistische Phasen, die jedoch durch andere Personen oder Ereignisse zerstreut werden konnten.
2 Patient war meist sehr pessimistisch in Bezug auf seine nahe Zukunft.
3 Patient sah keinerlei Hoffnung für sich und seine Lage in der Zukunft.

18. Selbstmordgedanken:

0 Patient hatte keinerlei Gedanken an Selbstmord oder Tod.
1 Patient empfand das Leben als leer oder nicht lebenswert.
2 Patient dachte mehrfach während der Woche für einige Minuten an Selbstmord oder Tod.
3 Patient dachte täglich ernsthaft an Selbstmord oder Tod, machte entsprechende Pläne oder versuchte Selbstmord zu begehen.

19. Interesse/Aktivitäten:

0 Die Interessen des Patienten für andere Menschen und Aktivitäten waren unverändert.
1 Patient merkte eine Verminderung von früheren Interessen und Aktivitäten.
2 Patient bewahrte nur noch ein oder zwei frühere Interessen.
3 Patient zeigte so gut wie kein Interesse mehr für frühere Aktivitäten.

20. Energie/Ermüdung:

0 Die Energie des Patienten war unverändert.
1 Patient ermüdete leichter als gewöhnlich.
2 Patient musste sich sehr anstrengen, um mit alltäglichen Aktivitäten zu beginnen und sie durchzuführen.
3 Patient war aufgrund seiner Energielosigkeit nicht in der Lage die meisten alltäglichen Dinge zu erledigen.

21. Vergnügen, Genuss (außer sexuellen Aktivitäten):

0 Patient beteiligte sich in gewohnter Weise an erfreulichen Aktivitäten und genoss sie.
1 Patient empfand bei erfreulichen Aktivitäten nicht die gewohnte Freude.
2 Patient empfand selten Freude egal bei welchen Aktivitäten.
3 Patient war unfähig, egal bei welchen Aktivitäten überhaupt Freude oder Genuss zu erleben.

22. Sexuelles Interesse:

0 Patient hatte ein unverändertes Interesse oder Freude an Sex.
1 Patient hatte ein fast normales Interesse bzw. hatte etwas Freude an Sex.
2 Patient hatte wenig Verlangen nach bzw. empfand selten Freude an Sex.
3 Patient war völlig uninteressiert an Sex bzw. hatte keine Freude daran.

23. Psychomotorische Hemmung:

0 Patient zeigte eine normale Geschwindigkeit beim Denken, Sprechen und Gestikulieren.
1 Patient empfand verlangsamtes Denken, und die Modulation der Stimme war herabgesetzt.
2 Patient benötigte meistens mehrere Sekunden um auf die meisten Fragen zu antworten; berichtete über verlangsamtes Denken.
3 Patient reagierte auf die meisten Fragen erst nach ausdrücklicher Aufforderung.

24. Psychomotorische Agitiertheit:

0 Der Denkvorgang des Patienten war nicht beschleunigt. Seine Gedanken waren geordnet. Gestik und Mimik waren unauffällig.
1 Patient war unruhig, knetete seine Hände und ging oft hin und her.
2 Patient fühlte sich getrieben und/oder war motorisch unruhig.
3 Patient konnte nicht sitzenbleiben. Lief mit oder ohne Erlaubnis umher.

25. Somatische Beschwerden:

0 Patient hatte kein Gefühl einer bleiernen Schwere in den Beinen und Armen oder Schmerzen.
1 Patient klagte über gelegentliche Kopf-, Bauch-, Rücken- oder Gelenkschmerzen, die ihn jedoch nicht behinderten.
2 Die genannten Beschwerden waren während der meisten Zeit vorhanden.
3 Die genannten Beschwerden führten zu einer Funktionseinschränkung.

26. Vegetative Erregung:

0 Patient berichtete nicht über Herzrasen, Schwitzen, Tremor, verschwommenes Sehen oder Hitzewallungen, Kälteschauer, Ohrengeräusche/Ohrensausen, Brustschmerzen oder Atemnot.
1 Die genannten Symptome waren nur leicht und zeitweilig vorhanden.
2 Die genannten Symptome waren mäßig stark und während mehr als der Hälfte der Zeit vorhanden.
3 Die genannten Symptome behinderten die Funktionen des Patienten.

27. Panik/Phobische Symptome:

0 Patient hatte keine Anzeichen von Panik oder phobischen Symptomen.
1 Patient hatte Zustände mit wenig ausgeprägter Panik oder phobischer Symptomatik, die ihn jedoch nicht weiter behinderten oder sein Verhalten beeinflussten.
2 Patient zeigte deutliche Zeichen einer Panik oder einer phobische Symptomatik, die sein Verhalten veränderten ohne ihn jedoch zu behindern.
3 Patient hatte mindestens einmal in der Woche lähmende Panikanfälle oder schwere Phobien, die zu völligem und regelmäßigem Vermeiden kritischer Situationen führten.

28. Verdauungsbeschwerden:

0 Der Stuhlgang des Patienten war unverändert.
1 Patient hatte gelegentlich Verstopfung und/oder leichten Durchfall.
2 Patient litt die meiste Zeit an Verstopfung und/oder Durchfall, die jedoch seine Funktionsfähigkeit nicht beeinträchtigten.
3 Patient litt wiederholt an behandlungsbedürftiger Verstopfung und/oder Durchfall mit Funktionseinschränkung.

29. Persönliche Beziehungen:

0 Patient hat sich durch andere nicht schnell abgewiesen, zurückgesetzt, kritisiert oder verletzt gefühlt.
1 Patient fühlte sich gelegentlich durch andere abgewiesen, zurückgesetzt, kritisiert oder verletzt.
2 Patient fühlte sich oft durch andere abgewiesen, zurückgesetzt, kritisiert oder verletzt, was seine soziale und Arbeitsfunktion nur leicht beeinflusste.
3 Patient fühlte sich oft durch andere abgewiesen, zurückgesetzt, kritisiert oder verletzt, was seine soziale und Arbeitsfunktion stark beeinträchtigte.

30. Schweregefühl/Körperliche Energie::

0 Patient hatte kein Gefühl einer bleiernen Körperschwere oder mangelnder Energie.
1 Patient hatte gelegentlich das Gefühl einer bleiernen Körperschwere und mangelnder Energie, jedoch ohne Beeinträchtigung von Arbeit, Schule oder Aktivitätsniveau.
2 Patient hatte mehr als die Hälfte der Zeit das Gefühl einer bleiernen Körperschwere (Mangel an körperlicher Energie).
3 Patient hatte die meiste Zeit das Gefühl einer bleiernen Schwere (Mangel an körperlicher Energie), d.h. mehrere Stunden am Tag und mehrere Tage pro Woche.

Gesamtscore:
